# Impairment in delay discounting in schizophrenia and schizoaffective disorder but not primary mood disorders

**DOI:** 10.1038/s41537-018-0050-z

**Published:** 2018-05-28

**Authors:** Hannah E. Brown, Kamber L. Hart, Leslie A. Snapper, Joshua L. Roffman, Roy H. Perlis

**Affiliations:** 1 Department of Psychiatry, Center for Quantitative Health, Massachusetts General Hospital and Harvard Medical School, Boston, MA USA; 2 Schizophrenia Clinical and Research Program, Massachusetts General Hospital, Boston, MA USA; 3 Department of Psychiatry, Massachusetts General Hospital and Harvard Medical School, Boston, MA USA

## Abstract

A measure of planning and impulse control, the delay-discounting (DD) task estimates the extent to which an individual decreases the perceived value of a reward as the reward is delayed. We examined cross-disorder performance between healthy controls (*n* = 88), individuals with bipolar disorder (*n* = 23), major depressive disorder (*n* = 43), and primary psychotic disorders (schizophrenia and schizoaffective disorder; *n* = 51) on the DD task (using a $10 delayed larger reward), as well as the interaction of DD scores with other symptom domains (cognition, psychosis, and affect). We found that individuals with schizophrenia and schizoaffective disorder display significantly greater rates of discounting compared to healthy controls, while individuals with a primary mood disorder do not differ from healthy controls after adjustment for IQ. Further, impairment in working memory is associated with higher discounting rates among individuals with schizophrenia and schizoaffective disorder, but cognitive dysfunction alone does not account for the extent of impairment in DD. Taken together, these results suggest an impaired ability to plan for the future and make adaptive decisions that are specific to individuals with psychotic disorders, and likely related to adverse functional outcomes. More generally, this work demonstrates the presence of variation in impulsivity across major psychiatric illnesses, supporting the use of a trans-diagnostic perspective.

## Impulsivity in schizophrenia: Seeking immediate reward

Patients with schizophrenia find it harder to delay gratification compared with patients diagnosed with other psychiatric disorders. Hannah Brown and colleagues in Roy Perlis’ lab at the Center for Quantitative Health at Massachusetts General Hospital, Boston, compared the performance of patients with bipolar disorder, major depressive disorder, schizophrenia, and healthy controls on a task that measures impulsivity. The delay discounting (DD) task assesses individuals’ ability to put off immediate pleasures for greater enjoyments later and is indicative of decision-making and planning behaviors. They found that certain aspects of cognitive dysfunction were associated with an increased preference for immediate rewards and that, even after adjusting for IQ, patients with schizophrenia showed significantly greater discounting rates compared with the other study participants. This variation in a specific measure of impulsivity suggests that the DD task could be used to better define aspects of impulsive behaviors across psychiatric disorders.

## Introduction

There is increased interest in understanding the aspects of psychopathology that cross traditional psychiatric diagnostic categories.^1^ One such trait that occurs trans-diagnostically is impulsivity. Impulsive behavior can be characteristic of a range of psychiatric diagnoses, including bipolar disorder (BPAD), substance use disorders, attention-deficit hyperactivity disorder, and psychotic disorders. Impulsivity as a concept spans multiple dimensions of behavior and cognition, including motivation, information processing, and response output.^2^ One validated measure of impulsivity is an individual’s behavior toward a certain reward, captured in the delay-discounting (DD) task. “Discounting” refers to the extent to which the perceived value of a reward is decreased when it is delayed. Here, impulsivity is characterized by choosing a smaller, immediate reward instead of a larger, delayed reward. The steepness of the discounting rate determines the degree of discounting (e.g., a lower rate indicates a greater willingness to wait for a larger but delayed reward). The dimensional nature of psychopathology can also be examined using the framework of the National Institute of Mental Health Research Domain Criteria (RDoC). Here, the DD task falls within both the positive valence (reward/motivation) and cognitive (inhibition/suppression) RDoC domains.^1^

Performance on the DD task may be informative about brain reward circuitry, and may have direct clinical implications for patients with primary psychotic disorders such as schizophrenia and schizoaffective disorder (SCZ/SCAD), or primary mood disorders such as BPAD or major depressive disorder (MDD), as decreased preference for future rewards may directly impact their ability to appreciate the necessity of long-term treatment or planning for the future.^2–5^ In addition to planning behaviors, for a subset of acutely suicidal depressed patients, DD task performance may indicate increased risk for suicidal behavior.^6^

Previous work in single-disease cohorts has demonstrated an elevated DD rate in individuals with SCZ/SCAD, BPAD, and MDD when compared to healthy control individuals (HCs).^7–9^ While healthy individuals with a less-steep rate of discounting have better performance on working-memory tasks, less is known about the effects of cognition on DD performance across diagnostic groups.^10^ In the present study, we sought to understand how impulsivity, as measured by the DD task, varies across psychiatric disorders. We compared DD behaviors among psychiatric diagnoses (SCZ/SCAD, BPAD, and MDD) and between each individual psychiatric diagnosis and HCs. Then, in an effort to understand how variation in impulsivity correlates with other symptoms, we also characterized the interaction between impulsivity and two other symptom domains, cognition and depression. While the domains are intended to be orthogonal, there are overlaps in brain systems underlying them, and further examination of domain interactions is of clinical relevance.^11^ Based on previous work, we hypothesized that (1) individuals with SCZ/SCAD, MDD, and BPAD would exhibit an elevated rate of DD compared to HCs, and (2) measures of cognition, in particular measures of executive function and strategy/planning behaviors, would also correlate with the rate of DD.

## Results

### Demographic and clinical phenotypes

Demographic and clinical characteristics across diagnostic groups are described in Table 1. The distribution of race and gender did not differ significantly across diagnostic groups; however, the HC cohort was on average younger than those in the diagnostic categories. Among the SCZ/SCAD population, the mean Positive and Negative Syndrome Scale (PANSS) total score was 70.8 (SD = 14.7), indicating a moderate degree of severity of psychotic symptoms. Both the BPAD and MDD groups (but not the HC or SCZ groups) scored on average in the moderate depression range. There was no difference in depression severity based on the Inventory of Depressive Symptomatology—Self-Report (IDS-SR) score between the BPAD and MDD group, and HCs had few or no depressive symptoms. There was a significant difference between diagnostic groups by intelligence quotient (IQ); the SCZ/SCAD group, and BPAD group had significantly lower mean IQ compared to HCs, and the SCZ/SCAD group had lower IQ scores than individuals with MDD in Tukey post hoc comparisons.

**Table 1:** Clinical characteristics and demographic information

	SCZ/SCAD	BPAD	MDD	HCs	Test statistic	*p*	Post hoc comparisons
	(*n* = 51)	(*n* = 23)	(*n* = 43)	(*n* = 88)			
Age (mean (SD))	43.6 (12.7)	46.6 (12.4)	44.3 (13.6)	35.6 (10.4)	*p* = 9.59	<0.001*	HC < BP, SCZ/SCAD, and MDD
Sex (male) (%)	33 (64.7)	12 (52.2)	22 (51.2)	40 (45.5)	*χ*^2^ = 4.82	0.186	
*Race*					*χ*^2^ = 7.95	0.9259	
Caucasian (%)	37 (72.5)	18 (78.3)	35 (81.4)	63 (71.6)			
African American (%)	6 (11.8)	4 (17.4)	4 (9.3)	10 (11.4)			
Asian (%)	5 (9.8)	1 (4.3)	3 (7.0)	10 (11.4)			
American Indian/Alaskan (%)	2 (3.9)	0 (0.0)	1 (2.3)	1 (1.1)			
Other (%)	1 (2.0)	0 (0.0)	0 (0.0)	4 (4.5)			
Current substance abuse— (%)	0 (0.0)	1 (4.3)	2 (4.7)	0 (0.0)	*χ*^2^ = 6.42	0.093	
Current anxiety disorder (%)	12 (23.5)	14 (60.9)	22 (51.2)	0 (0.0)	*χ*^2^ = 63.36	<0.001*	HC < MDD, BPAD, and SCZ/SCAD; SCZ/SCAD < BPAD; and SCZ/SCAD < MDD
Current alcohol use disorder (%)	1 (2.0)	0 (0.0)	4 (9.3)	0 (0.0)	*χ*^2^ = 11.34	0.01*	HC < MDD
Current antipsychotic use (%)	45 (88.2)	15 (65.2)	9 (20.9)	0 (0.0)	*χ*^2^ = 126.06	<0.001*	HC < MDD, BPAD, and SCZ/SCAD; MDD < BPAD; MDD < SCZ/SCAD; and BPAD < SCZ/SCAD
Current mood stabilizer use (%)	13 (25.5)	9 (39.1)	6 (14.0)	0 (0.0)	*χ*^2^ = 32.63	<0.001*	HC < MDD, BPAD, and SCZ/SCAD
Current antidepressant use (%)	22 (43.1)	15 (65.2)	17 (39.5)	0 (0.0)	*χ*^2^ = 73.44	<0.001*	HC < MDD, BPAD, and SCZ/SCAD
Smoking status (%)	13 (25.5)	7 (30.4)	10 (23.3)	3 (3.4)	*χ*^2^ = 20.27	<0.001*	HC < MDD, BPAD, and SCZ/SCAD
*Clinical measures*	*Mean (SD)*	*Mean (SD)*	*Mean (SD)*	*Mean (SD)*			
PANSS positive	16.04 (6.2)						
PANSS negative	22.8 (5.2)						
PANSS general	31.9 (6.7)						
PANSS total score	70.8 (14.7)						
IDS-SR	16.5 (12.1)	30.2 (17.5)	26.9 (15.5)	4.0 (3.7)	*p* = 58.78	<0.001*	HC < BPAD, SCZ/SCAD, and MDD; SCZ/SCAD < BPAD, MDD
*Cognitive measures*	*Mean (SD)*	*Mean (SD)*	*Mean (SD)*	*Mean (SD)*			
IQ	92 (14.1)	96 (18.7)	105.8 (18)	107.7 (15.5)	*p* = 12.32	<0.001*	HC > SCZ/SCAD, BPAD; SCZ/SCAD < MDD
AST	83.81 (13.90)	83.24 (18.76)	91.67 (8.00)	95.60 (4.63)	*p* = 17.82	<0.001*	HC > BPAD, SCZ/SCAD; MDD > BP, SCZ/SCAD
SWM	39.19 (24.23)	40.05 (31.59)	33.76 (24.42)	16.19 (17.07)	*p* = 15.39	<0.001*	HC < BPAD, MDD, and SCZ/SCAD
PAL	50.14 (51.59)	64.76 (80.19)	37.60 (50.15)	12.39 (27.69)	*p* = 11.38	<0.001*	HC < BPAD, SCZ/SCAD
Ln(*k*)	–3.65 (3.55)	–3.57 (2.77)	–4.94 (2.46)	–5.51 (2.12)	*p* = 6.76	<0.001*	HC < BPAD, SCZ/SCAD

*SCZ/SCAD* schizophrenia/schizoaffective disorder, *BPAD* bipolar affective disorder, *MDD* major depressive disorder, *HC* healthy control, *AST* attention switching task, *SWM* spatial working memory, *PAL* paired associates learning, *IQ* intelligence quotient, *PANSS* Positive and Negative Syndrome Scale, *IDS-SR* Inventory of Depressive Symptomatology – Self-Report

On average, each participant completed 183.2 trials (SD = 43.2). In repeated measures analysis of variance (ANOVA), there was no effect of diagnosis on the number of trials administered, and there was no interaction between trials per time point and diagnostic category (Supplemental Fig. 1). There was a significant effect of time point on the number of trials administered. Participants required fewer trials to reach an indifference point for the first two time points, as compared to all other time points. The average number of trials by time point and by diagnostic group, as well as the results of the repeated measures ANOVA and post hoc comparisons, are shown in Supplemental Fig. 1. Sixty-five participants (32%) with inconsistent discounting curves were excluded from the primary analysis based on the criteria discussed in the Data analysis section: 20 individuals with SCZ/SCAD, seven individuals with BPAD, 11 individuals with MDD, and 27 HCs. In general, excluded individuals were more likely to smoke and be taking psychiatric medication, but otherwise did not differ significantly from included individuals in diagnosis or other demographic features (Supplemental Table 1). Numerically, excluded individuals also had lower IQ, although this difference was not statistically significant (Supplemental Table 1). A *χ*^2^ test of homogeneity determined that diagnostic groups did not significantly differ in the proportion of inconsistent responders (*χ*^2^(2, *N* = 205) = 0.73, *p* = 0.69).

**Fig. 1:**
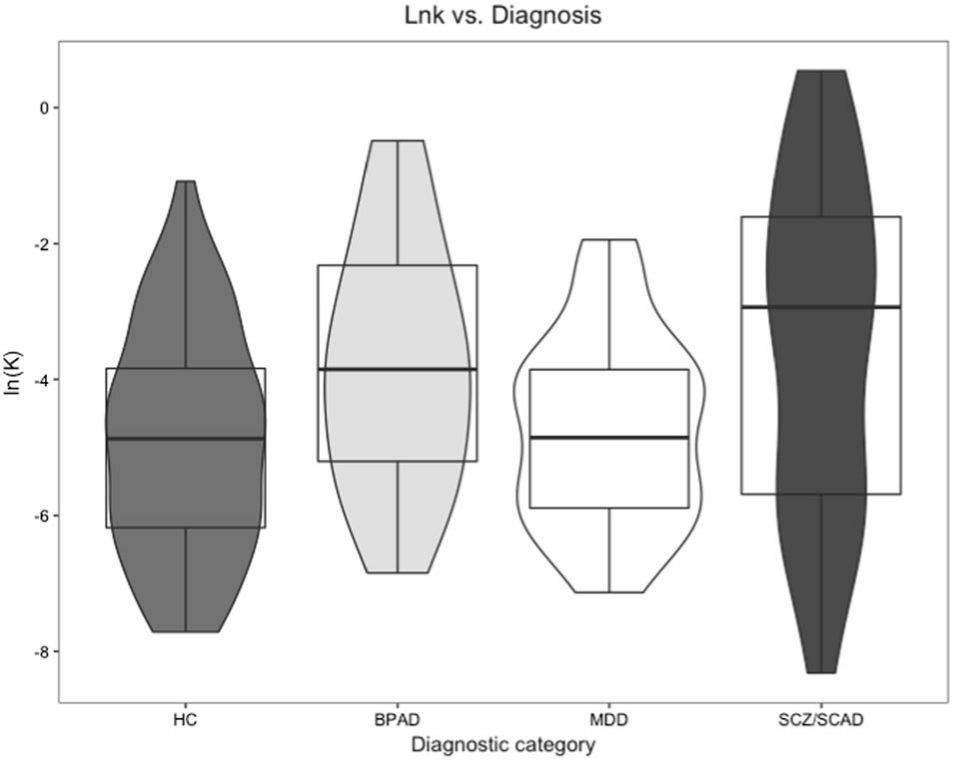
Distribution of the rate of delay discounting across the diagnostic category

After excluding individuals with inconsistent indifference points, we analyzed the differences in rates of DD between diagnostic categories using a one-way ANOVA and Tukey post hoc comparisons. There was a significant effect of diagnosis (*F*(3136) = 4.897, *p* = 0.003), and in post hoc comparisons, individuals with SCZ/SCAD had steeper discounting rates (ln*k*), as compared to HCs and individuals with MDD (*p* *=* 0.004 and *p* = 0.043, respectively), but there was no difference compared to individuals with BPAD (Supplemental Table 2). Next, we examined the effects of diagnosis on ln*k* in unadjusted models, as well as the unadjusted effects of our covariates (age, gender, medication use (antidepressants, antipsychotics, and mood stabilizers), smoking, and IQ; Table 2, left). In the unadjusted models, there was a significant effect of SCZ/SCAD diagnosis and BPAD diagnosis on the rate of DD, compared to HCs, with both diagnostic groups having steeper rates of discounting. There was no significant difference in DD rates between the MDD group and HCs. Univariate models also identified significantly steeper discounting rates due to age and current antipsychotic use, and a significantly less-steep discounting rate due to IQ (Table 2, left). Subsequently, we created an adjusted model to examine the adjusted effect of diagnosis on ln*k* while controlling for the aforementioned covariates (Table 2, right). In the adjusted model, IQ was a significant predictor of the rate of discounting: a one-point increase in IQ corresponded with a 0.029 decrease in ln*k* (or, for every one standard-deviation increase in IQ (15 points), ln*k* decreases by 0.44). Additionally in the adjusted model (taking into account the effects of IQ), only a diagnosis of SCZ/SCAD corresponded with a steeper rate of discounting compared to HCs (Table 2, right; Fig. 1).

**Table 2:** Unadjusted and adjusted models of delay-discounting rate among consistent participants

	Unadjusted models			Adjusted model		
	*β*	*T*-value	*p*	*β*	*T*-value	*p*
BPAD	1.088	2.122	0.036*	0.751	1.084	0.281
MDD	0.168	0.420	0.675	−0.044	−0.088	0.930
SCZ/SCAD	1.391	3.453	0.001*	1.437	2.090	0.039*
Age	0.033	2.610	0.010*	0.018	1.168	0.245
Sex	0.379	1.182	0.239	−0.018	−0.053	0.958
Antipsychotic Tx	1.004	2.941	0.004*	−0.722	−1.207	0.230
Mood stabilizer Tx	0.795	1.850	0.066	0.304	0.628	0.531
Antidepressant Tx	0.569	1.701	0.091	0.221	0.511	0.610
Smoking status	0.501	0.990	0.324	−0.521	−0.964	0.337
IQ	−0.038	−4.214	<0.001*	−0.029	−2.636	0.009*

*SCZ/SCAD* schizophrenia/schizoaffective disorder, *BPAD* bipolar affective disorder, *MDD* major depressive disorder, *HC* healthy control, *AST* attention switching task, *SWM* spatial working memory, *IQ* intelligence quotient, *tx* treatment

*p < 0.05

To better understand the impact of cognition on DD, and thus the extent to which one measure of impulsivity is orthogonal with cognition, we next examined the relationship between DD rate and three neuropsychological tasks (spatial working memory (SWM), paired associates learning (PAL), and attention-switching task (AST)) by replacing IQ in our adjusted model with outcome measures of these three tasks (Table 3). Within this model, there was a steeper rate of DD for both SCZ/SCAD diagnosis and the number of errors on the SWM task. Additionally, antipsychotic use was associated with a less-steep rate of discounting. There was no significant effect of PAL or AST performance on the rate of DD in any group. Thus, only a diagnosis of SCZ/SCAD remained a significant predictor of the steeper rate of DD after accounting for the effects of SWM and antipsychotic use.

**Table 3:** Adjusted model of delay-discounting rate including cognitive measures

	*β*	*T*-value	*p*
BPAD	0.956	1.315	0.191
MDD	−0.192	−0.387	0.699
SCZ/SCAD	1.879	2.710	0.008*
Age	0.008	0.496	0.621
Sex	0.090	0.259	0.796
Antipsychotic Tx	−1.263	−1.997	0.048*
Mood stabilizer Tx	0.346	0.695	0.488
Antidepressant Tx	0.170	0.393	0.695
Smoking status	−0.728	−1.282	0.202
SWM	0.029	2.976	0.004*
AST	−0.004	−0.221	0.826
PAL	0.001	0.166	0.869

*SCZ/SCAD* schizophrenia/schizoaffective disorder, *BPAD* bipolar affective disorder, *MDD* major depressive disorder, *HC* healthy control, *AST* attention switching task, *SWM* spatial working memory, *PAL* paired associates learning, *tx* treatment

*p < 0.05

Next, we repeated our adjusted model (including the more global measure of cognitive functioning, IQ), controlling for comorbid psychiatric diagnoses, including anxiety disorders, alcohol abuse/dependence, and substance abuse/dependence. The results of this analysis are presented in Supplemental Table 3. Again, individuals with SCZ/SCAD showed a steeper rate of discounting, and an increase in IQ was associated with a significant decrease in the rate of discounting.

Finally, we sought to understand the relationship between symptom severity and DD results, and performed separate analyses within each diagnostic group incorporating diagnosis-specific clinical measures (Table 4). Within the SCZ/SCAD model, there was no effect of symptom severity on DD (as measured by PANSS negative, positive, and general scores); however, an increased score on the SWM task (increased number of errors between each round) corresponded with a significant increase in the rate of DD within the SCZ/SCAD group after controlling for clinical measures. Within the MDD group model, an increased score on the IDS-SR (and thus greater depressive symptomatology) corresponded to a less-steep rate of discounting. Within the BPAD group model, depression severity did not have an effect on the rate of discounting. Finally, among HCs, only an increase in the SWM score significantly corresponded to a steeper rate of DD.

**Table 4:** Delay-discounting rate and symptom severity within diagnostic groups

	SCZ/SCAD			BPAD			MDD			HC		
	*β*	*T*-value	*p*	*β*	*T*-value	*p*	*β*	*T*-value	*p*	*β*	*T*-value	*p*
Smoking status	−0.284	−0.197	0.846	−0.971	−0.633	0.542	0.040	0.061	0.952	−1.466	−0.803	0.425
Age	−0.038	−0.779	0.444	0.063	1.222	0.253	0.029	1.195	0.243	0.002	0.077	0.939
Sex	−0.115	−0.101	0.920	0.830	0.673	0.518	0.148	0.289	0.775	0.531	1.188	0.240
IDS-SR score	—	—	—	0.060	1.633	0.137	−0.042	−2.895	0.008*	−0.063	−1.093	0.279
SWM	0.065	2.324	0.030*	0.003	0.148	0.885	0.003	0.210	0.835	0.033	2.507	0.015*
PANSS (negative)	0.141	1.269	0.218	—	—	—	—	—	—	—	—	—
PANSS (positive)	0.018	0.115	0.909	—	—	—	—	—	—	—	—	—
PANSS (general)	−0.138	−1.088	0.289	—	—	—	—	—	—	—	—	—

*SCZ/SCAD s*chizophrenia/schizoaffective disorder, *BPAD* bipolar affective disorder, *MDD* major depressive disorder, *HC* healthy control, *PANSS* Positive and Negative Syndrome Scale, *IDS-SR* Inventory of Depressive Symptomatology – Self-Report

*p < 0.05

## Discussion

In this study, we looked trans-diagnostically at one measure of impulsivity and its interaction with other symptom domains. Individuals with SCZ/SCAD demonstrated a greater rate of DD compared to individuals with MDD and HCs—that is, those with psychotic illness prefer smaller and sooner rewards compared to larger and later rewards. Further, in adjusted models, we found that performance on certain cognitive tasks also influenced DD; individuals with more impairment on the SWM task demonstrate a steeper rate of discounting, and individuals with a lower IQ likewise have a steeper DD rate. Current treatment with antipsychotic medication did not have a significant effect on the rate of DD in adjusted models. Among individuals with MDD, a greater level of depressive symptomatology was predictive of a less-steep rate of discounting (decreased ln*k*), but no such relationship was observed among other mood disorders or HCs. These findings underscore the important point that, despite efforts to isolate individual areas of neuropsychological function as orthogonal domains, there is correlation among cognitive paradigms across psychiatric disorders.

Our finding that individuals with SCZ/SCAD exhibit greater rates of DD compared to HCs extends previous reports.^7,12–14^ A steeper rate of discounting among psychotic patients indicates an impaired ability to plan for future consequences and make adaptive decisions, which can have significant functional consequences in everyday life (e.g., saving money, planning meals, and obtaining shelter). Within the SCZ/SCAD group as well as the HC group, an increase in SWM errors correlates with a higher rate of DD, findings that are, in general, in keeping with previous literature examining working memory in relation to DD.^7,10^ SWM is known to be impaired in individuals with SCZ, so it stands to reason that impairment in SWM would covary with the rate of discounting within the SCZ/SCAD cohort.^15^ SWM demands both the ability to retain and manipulate visuospatial information, as well as a degree of strategy and planning behaviors, which are necessary components for optimal decision-making in the DD task. Additionally, within this model, taking antipsychotic medication was associated with a less-steep rate of discounting. We postulated that antipsychotic treatment could affect performance on the DD task; antipsychotic medications modulate dopaminergic brain circuits, and decreased dopamine receptor (including D2) availability is associated with an increased rate of DD.^16^ However, in this study, current treatment with antipsychotic medication resulted in a less-steep rate of DD in adjusted models controlling for cognitive domains. However, we did not examine specific antipsychotic dosing, so we cannot exclude the possibility that higher doses of antipsychotic medication with greater D2 blockade would impact DD rate differently (i.e., cause a steeper rate of DD).

Looking trans-diagnostically, two other cognitive measures (PAL, a measure of visual memory and learning, and AST, a measure of executive functioning) do not affect performance on the DD task. We postulated that performance on AST would affect performance on the DD task, given that the DD task requires planning behaviors. However, as discussed, the DD task is not purely an executive-functioning measure, and thus is not significantly affected by AST performance. The DD task does not allow for error processing or learning, since the participant is instructed to respond instinctively; thus, the task measures one component of impulsivity.^17,18^ For this reason, we did not expect PAL to influence DD performance, since learning should not play a role in task performance. Therefore, within working memory, impairments in SWM specifically relate to increased rates of DD, as compared to measures of executive functioning or learning.

Within the SCZ/SCAD group, we did not detect any association between the severity of clinical symptoms (as measured by the PANSS) and the rate of discounting, which is consistent with some previous findings.^7^ Interestingly, studies in first-degree family members of individuals with SCZ/SCAD (depressed type) have not identified abnormalities in DD, which might suggest that this is a state rather than a trait-dependent phenomenon.^14^ While we did examine the overall PANSS negative scores, one component of motivational negative symptoms not directly addressed in this study is effort-based decision-making, i.e., the effort an individual expends for a particular reward. Thus, as the reward increases, so should the effort expended to obtain the reward.^19^ Individuals with SCZ/SCAD may be less motivated to pursue a reward or cannot comprehend the structure of the reward itself, which may directly affect their performance on the DD task.

In contrast to the SCZ/SCAD cohort, we did not find a significant difference in the rate of DD between either MDD or BPAD individuals and HCs. This lack of difference may be indicative of the state of the participant at the time of testing. While manic patients may have a greater rate of DD, our cohort of BPAD was predominantly depressed, likely explaining the lack of difference in the discounting rate between both MDD and BPAD and HCs.^7^ However, within just the MDD group, more severe depressive symptoms were associated with a less-steep rate of DD. The presence of moderate depression may prevent individuals from appreciating the benefit of any immediate gratification, causing them to put off future rewards. However, it is possible that with a larger future reward (e.g., >$10), depressed individuals with MDD will discount at higher rates, as noted in Pulcu et al.^8^ Additionally, MDD symptoms are heterogeneous, and it is possible that different symptoms affect DD performance to varying extents. For example, those individuals who display more anhedonia may have a less-steep rate of discounting compared to those who are less anhedonic.^20^ Within the BPAD cohort, depressive symptomatology did not correlate with the rate of DD; this may simply reflect the modest size of the BPAD cohort yielding consistent performance (*n* = 16).

One potential limitation to our study is the reward size offered in the DD task. Participants chose between the sooner smaller reward (SSR) and the larger later reward (LLR), which was fixed at $10. For some participants, this monetary amount may not be significantly large enough to delay, and thus they may choose the SSR, regardless of amount. To this point, Wing et al.^21^ found that reward size influenced the rate of discount (i.e., a smaller reward resulted in a higher delay-discounting rate). Another limitation is that there was no assessment of manic symptoms using a specific clinical scale (e.g., Young Mania Rating Scale); rather, we relied solely on the Structured Clinical Interview for DSM-IV (SCID) assessment of manic symptoms. It is possible that if we used such a specialized scale, more manic symptoms would be apparent, and perhaps, a difference could be seen between a subgroup of BPAD patients and HCs.

A third potential limitation is the exclusion of individuals who are deemed inconsistent, based on the relationship between the indifference points and the LLR. For example, a consistent choice of the SSR is labeled inconsistent because the last indifference point is not less than the first indifference point by at least 10% of the LLR, and thus does not fit the hyperbolic model. However, this pattern of choice may in fact be indicative of the underlying psychopathology and disease state; for this reason, in contrast to many prior studies, we examine and detect associations with exclusion, and suggest that future studies should do the same. Furthermore, as noted in previous studies, the monetary reward in the DD task is hypothetical and thus potentially less impactful than real payment; however, even hypothetical tasks have been shown to produce similar discounting rates when compared to tasks using real money.^22^

Finally, even though prior literature indicates an effect of substance use on DD (and in particular cigarette smoking), low rates of current substance use in our population preclude direct examination of effects other than nicotine use.^23^ Recent work demonstrates that elevated DD rate among SCZ/SCAD persists, regardless of smoking behaviors, which is consistent with our results.^14^

Taken as a whole, the present study illustrates significant variation in a specific measure of impulsivity across major psychiatric disorders, supporting the use of such measurement even in disorders where reward processing per se is not understood to be the primary area of dysfunction. This work also underscores the challenges in multidimensional assessment of psychopathology: despite ongoing efforts to isolate individual symptom measurements, many paradigms require intactness of multiple symptom domains. Here, cognition (measured grossly by IQ, and more specifically by SWM) associates with performance on the DD task. Understanding the relationship between symptom domains may allow for further clinical characterization using dimensional measures, and may provide the opportunity for more targeted therapeutic strategies. Considering the impact of impulsive behavior on contributors to adverse psychiatric outcomes, including substance abuse, treatment nonadherence, impaired decision-making, and self-injury, further investigation of this behavior—and the ways in which it interacts with diagnosis and other symptoms—is warranted.

## Methods

### Participants

Participants were recruited from the outpatient clinical setting, as part of a systematic clinical assessment for a cellular biobanking study.^24^ The Partners Institutional Review Board approved the study protocol and informed consent procedure, and all studies were performed in accordance with relevant guidelines and regulations. All participants provided written informed consent. Participants ranged in ages from 20 to 65 years and represented four diagnostic categories: (1) 43 participants with MDD, (2) 51 participants with primary psychotic disorder (SCZ (*n* = 33) or SCAD (*n* = 18)), (3) 23 participants with BPAD, as well as (4) 88 HCs. An expert clinician confirmed the diagnosis for participants with psychiatric illness and the lack of diagnosis for HCs using the Structured Clinical Interview for DSM-IV (SCID)^25^ and the Mini International Neuropsychiatric Interview Version 5.0.0 (MINI).^26^ For those individuals with a primary psychotic disorder, psychotic symptoms were evaluated by a trained physician rater using the PANSS.^27^ There was no active symptom requirement for participation in the study. Smoking status was obtained by a self-report: participants reported their smoking status over the past 2 weeks, and “smokers” were classified as those who reported smoking between several days to nearly every day. Current alcohol use and other substance use disorders were obtained using the MINI. Current medication use was assessed with a questionnaire administered by the study clinician, confirming medication name, dose, and duration of the current treatment episode. Medications were confirmed by a query of electronic health records wherever available. The IDS-SR, a 30-item assessment of depression severity, was administered to all participants.^28^ As part of the cognitive battery described below, participants completed the Wechsler Abbreviated Scale of Intelligence second edition (WASI-II) to determine the full-scale IQ, using the vocabulary and matrix-reasoning subsections.^29^ HCs were defined as individuals having no Axis I psychiatric diagnosis by SCID and MINI, and no intellectual disability (defined by IQ <70). Participants were excluded if they had intellectual disability, or a comorbid neurologic illness, including Parkinson’s disease, multiple sclerosis, or Alzheimer’s disease based upon clinical interview and review of electronic health records.

### Neuropsychiatric battery

In addition to the WASI-II, all participants completed a computerized neuropsychiatric cognitive battery, the Cambridge Neuropsychological Test Automated Battery (CANTAB), which includes testing of specific cognitive domains.^30^ The present analyses focused on standard CANTAB measures, including SWM, PAL, and AST. For SWM, we analyzed the number of errors made between attempts, for PAL total errors, and for AST percent correct trials. These tasks were selected based on validity and ability to assess the relevant domains of cognition.

### Delay-discounting tasks

Each participant was administered a DD task.^17,31^ In this DD task, participants were given the choice between a SSR, and a set LLR of $10. Reward levels for the sooner smaller reward varied between 50 cents and $10. An example question is as follows: “would you rather have 3 dollars now or 10 dollars in 365 days?” The time points for the LLR ranged from immediately to 1 year (0, 2, 30, 180, and 365 days). Participants were instructed to respond instinctively, as is standard administration practice. As the participants answered questions, the program adjusted the amount of the SSR based on their previous responses, and the participant continued to answer until a point of indifference was reached for each time point. The indifference point is the value at which the participant is indifferent between the two choices.^17^

### Data analysis

All analyses were done using R software, version 3.2.1.^32^ Since there was a variable number of trials completed by a participant at each indifference point, we conducted repeated measures ANOVA to determine the effect of diagnostic category and time point on the number of trials administered. Subsequently, we performed post hoc pairwise comparisons between time points using paired two-sided *t* tests with Bonferroni corrections.

Then, in accordance with previously described methods, the indifference points for each of the five time points were calculated for each participant. As is standard in the literature, the five indifference points were used to determine a best-fit hyperbolic curve.^33^

$$V{\mathrm{p}} = \frac{A}{{1 + \left( {k \times D} \right)}}.$$

In this equation, *V*p is the discounted value of the reward, *A* is the value of the later larger reward ($10 in this study), *k* is the discounting rate for the hyperbolic curve, and *D* is the amount of time between the choice and the reward (delay).^34^ If the software could not validate an indifference point, then that point was not used to fit the curve, consistent with prior studies.^33^ The hyperbolic discounting rate for time, *k*, was determined using a nonlinear least-squares approach to fit the model. A larger *k* indicates a steeper rate of discounting (i.e., prefers smaller sooner rewards), and thus greater impulsivity. A smaller *k* indicates a less-steep rate of discounting (i.e., prefers larger later rewards). As in prior literature, we used the natural log (ln) of *k* to normalize the distribution of *k* for all subsequent analyses, since *k* has a strong positive skew.^7,17^

Individuals with inconsistent discounting curves—i.e., participants whose indifference points could not be fit using a hyperbolic curve—were identified according to the model proposed by Johnson and Bickel.^34^ Specifically, participants were designated as having inconsistent discounting curves if they (1) had an indifference point that increased by >20% of the LLR ($2 in this paradigm) or (2) if the last indifference point was not less than the first indifference point by at least 10% of the LLR ($1 in this paradigm).

Participants with inconsistent discounting curves were excluded from the main analyses because their data are nonsystematic and cannot be interpreted using a hyperbolic model.^34^ However, inconsistent performance could be the result of disease-associated cognitive dysfunction. Therefore, rather than simply omitting these individuals, we began by comparing excluded individuals to the remainder of the cohort. Multivariable logistic regression was used to compare individuals with inconsistent and consistent indifference points, both across and within diagnoses, examining the specific metrics relevant to each diagnostic category (PANSS for SCZ/SCAD; IDS-SR for BPAD and MDD). Additionally, a *χ*^2^ test of homogeneity was performed to determine if diagnostic groups were comparable in the rate of inconsistency. Assuming 20 subjects per group, with *α* = 0.05, power exceeds 80% to detect a minimum effect size of 0.38.

Subsequent analyses focused on the consistent (remaining) cohort. First, we performed a one-way ANOVA to compare the differences in DD rates between all diagnostic groups and performed pairwise post hoc comparisons of the discounting rate between diagnostic groups. Next, we assessed the effects of diagnosis on DD in unadjusted linear-regression models, as well as in an adjusted model incorporating age, sex, smoking status, current use of a psychiatric medication, cognitive measures (IQ and SWM, PAL, and AST), and depressive measures (IDS-SR score). We included the relevant psychiatric medications (antipsychotics, antidepressants, and non-antipsychotic mood stabilizers) in our adjusted models since use of all three medications was present in each of the diagnostic groups. Information about all classes of medication was obtained via a questionnaire administered by a study clinician. Additionally, we repeated the adjusted model, controlling for psychiatric comorbidities, including anxiety disorders, alcohol abuse/dependence, and substance abuse/dependence. We also compared DD performance within each individual diagnostic category (SCZ/SCAD, BPAD, and MDD) to DD performance in the HC group. In addition, to explore the impact of symptom severity on DD, we created linear models specific to each diagnostic category. These models included clinical assessments specific to the diagnostic category (PANSS scores for patients with SCZ/SCAD, and IDS-SR scores for patients with MDD and BPAD) and controlled for age, sex, smoking status, and SWM shown to have a significant effect in the previous model.

### Data availability

De-identified data will be available from the authors upon request.

## Electronic supplementary material

Supplemental Table 1

Supplemental Table 2

Supplemental Table 3

Supplemental Figure 1
