# Supplementary material for: Impairment in delay discounting in schizophrenia and schizoaffective disorder but not primary mood disorders
Source: NPJ Schizophr. 2018 May 28;4:9. doi: 10.1038/s41537-018-0050-z (PMC5972152; doi:10.1038/s41537-018-0050-z)
Supplement: Supplementary file 1 — Supplemental Table 1 [file 41537_2018_50_MOESM1_ESM.docx]

Supplemental Table 1: Adjusted odds ratio for inconsistency within demographic categories and clinical measures

|  | | |  |  |
| --- | --- | --- | --- | --- |
| Outcome | Adjusted Odds Ratio | 95 % Confidence Interval | | *p* |
| Age | 0.971 | 0.940 | 1.002 | 0.070 |
| Sex | 1.103 | 0.553 | 2.209 | 0.780 |
| BPAD | 0.843 | 0.165 | 4.009 | 0.833 |
| MDD | 0.886 | 0.286 | 2.568 | 0.827 |
| SCZ/SCAD | 0.505 | 0.113 | 2.063 | 0.352 |
| Antipsychotic Tx | 3.653 | 1.049 | 14.031 | 0.048 * |
| Mood Stabilizer Tx | 0.194 | 0.045 | 0.673 | 0.016 * |
| Antidepressant Tx | 0.355 | 0.129 | 0.922 | 0.037 * |
| Smoking Status | 5.174 | 1.908 | 15.078 | 0.002 * |
| IQ | 0.983 | 0.960 | 1.005 | 0.138 |

* *p* < 0.05; BPAD – Bipolar Affective Disorder, MDD – Major Depressive Disorder; SCZ/SCZD – Schizophrenia / Schizoaffective Disorder;

AST – Attention Switching Task, SWM – Spatial Working Memory; PAL – Paired Associates Learning
