# Supplementary material for: Impairment in delay discounting in schizophrenia and schizoaffective disorder but not primary mood disorders
Source: NPJ Schizophr. 2018 May 28;4:9. doi: 10.1038/s41537-018-0050-z (PMC5972152; doi:10.1038/s41537-018-0050-z)
Supplement: Supplementary file 2 — Supplemental Table 2 [file 41537_2018_50_MOESM2_ESM.docx]

Supplemental Table 2: Post-hoc Comparisons of ln*k* Between Diagnostic Groups within Consistent Responders

|  |  |  |  | Tukey’s HSD Comparisons - p values | | |
| --- | --- | --- | --- | --- | --- | --- |
| Group | *n* | Mean | *SD* | HC | BPAD | MDD |
| HC | 61 | -4.907 | 1.647 | - | - | - |
| BPAD | 16 | -3.819 | 1.842 | 0.151 | - | - |
| MDD | 32 | -4.740 | 1.386 | 0.975 | 0.356 | - |
| SCZ/SCAD | 31 | -3.517 | 2.451 | 0.004* | 0.950 | 0.043* |

* *p* < 0.05; BPAD – Bipolar Affective Disorder, MDD – Major Depressive Disorder; SCZ/SCZD – Schizophrenia / Schizoaffective Disorder;

AST – Attention Switching Task, SWM – Spatial Working Memory; PAL – Paired Associates Learning;

IDS-SR - Inventory of Depressive Symptomatology – Self-Report
