# Supplementary material for: Impairment in delay discounting in schizophrenia and schizoaffective disorder but not primary mood disorders
Source: NPJ Schizophr. 2018 May 28;4:9. doi: 10.1038/s41537-018-0050-z (PMC5972152; doi:10.1038/s41537-018-0050-z)
Supplement: Supplementary file 3 — Supplemental Table 3 [file 41537_2018_50_MOESM3_ESM.docx]

Supplemental Table 3:

|  | **β** | **T-value** | ***p*** |
| --- | --- | --- | --- |
| BPAD | 0.814 | 1.072 | 0.286 |
| MDD | 0.070 | 0.122 | 0.903 |
| SCZ/SCAD | 1.452 | 2.027 | 0.045 * |
| Age | 0.017 | 1.097 | 0.275 |
| Sex | 0.017 | 0.051 | 0.960 |
| Antipsychotic Tx | -0.645 | -1.044 | 0.299 |
| Mood Stabilizer Tx | 0.229 | 0.461 | 0.646 |
| Antidepressant Tx | 0.241 | 0.541 | 0.589 |
| Anxiety Disorder | -0.256 | -0.571 | 0.569 |
| Alcohol Abuse / Dependence | -0.328 | -0.172 | 0.864 |
| Drug Abuse / Dependence | 0.932 | 0.669 | 0.505 |
| Smoking Status | -0.446 | -0.806 | 0.422 |
| IQ | -0.028 | -2.537 | 0.013* |

* *p* < 0.05; BPAD – Bipolar Affective Disorder, MDD – Major Depressive Disorder; SCZ/SCZD – Schizophrenia / Schizoaffective Disorder;

AST – Attention Switching Task, SWM – Spatial Working Memory; PAL – Paired Associates Learning
