# Supplementary material for: Impairment in delay discounting in schizophrenia and schizoaffective disorder but not primary mood disorders
Source: NPJ Schizophr. 2018 May 28;4:9. doi: 10.1038/s41537-018-0050-z (PMC5972152; doi:10.1038/s41537-018-0050-z)
Supplement: Supplementary file 4 — Supplemental Figure 1 [file 41537_2018_50_MOESM4_ESM.pdf]

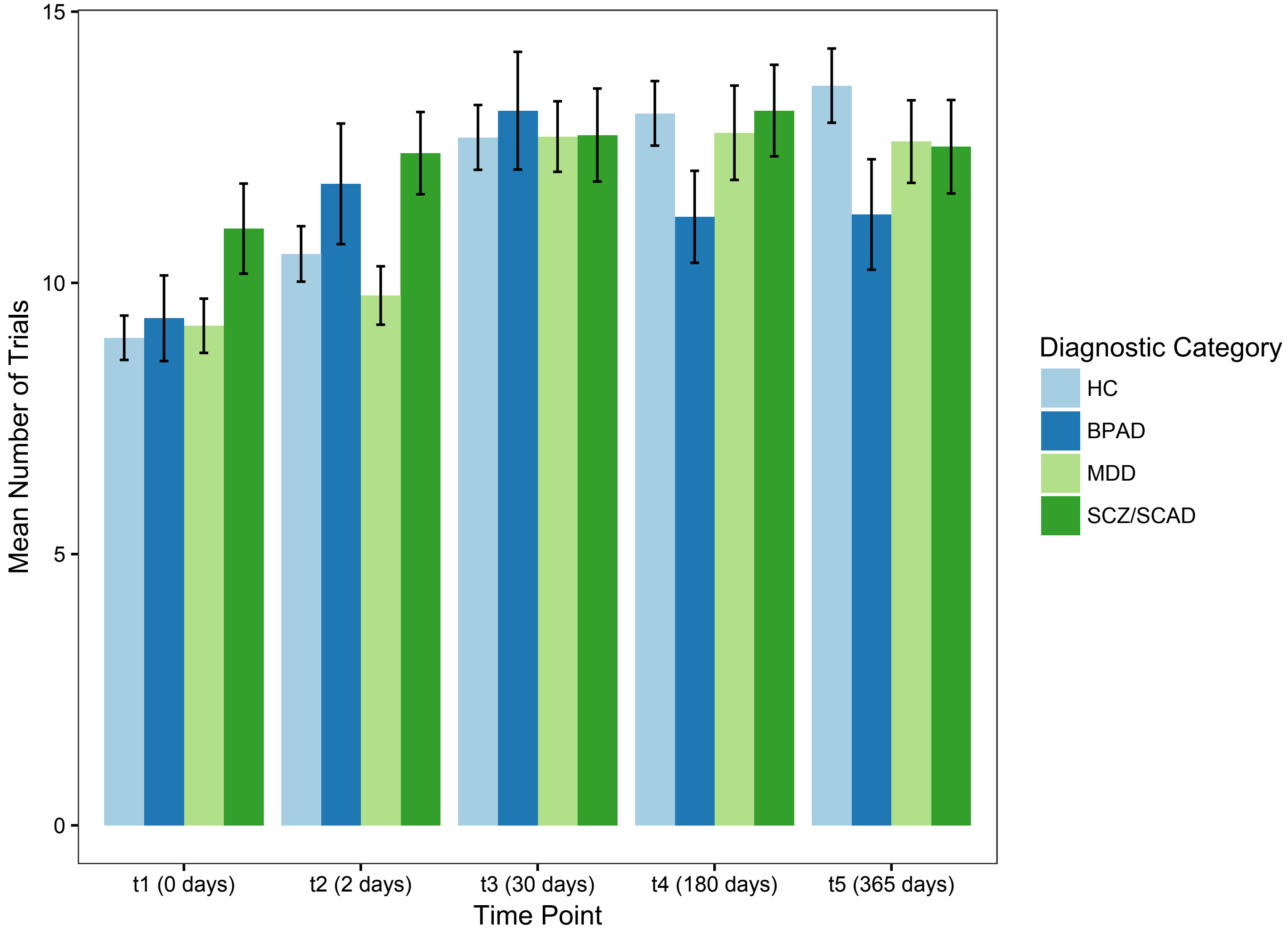

Repeated Measures ANOVA of Time Point and Diagnosis

	DF	F	MSE	p
**Diagnosis**	1, 203	0.7518155	34.461	0.387
**Time Point**	4,812	17.3685003	25.89513	0.000
**Diagnosis * Time Point**	4,812	1.6694189	25.895	0.155

Pairwise Comparisons Between Time Points Using Paired T-Tests and Bonferroni Adjustment

	t1	t2	t3	t4
**t2**	0.0119 *	-	-	-
**t3**	3.70×10^{-09} *	0.0042*	-	-
**t4**	7.60×10^{-11} *	0.0012*	1	-
**t5**	4.60×10^{-08} *	0.0054*	1	1

DF = Degrees of Freedom, MSE = Mean Squared Error, HC = Healthy Control, BPAD – Bipolar Disorder, MDD – Major Depressive Disorder, SCZ/SCAD – Schizophrenia / Schizoaffective Disorder, Error bars represent standard error.
